# Quality of Life in People with Type 2 Diabetes in Relation to Deprivation, Gender, and Age in a New Community-Based Model of Care

**DOI:** 10.1155/2011/613589

**Published:** 2011-06-21

**Authors:** Grace Lindsay, Kathryn Inverarity, Joan R. S. McDowell

**Affiliations:** ^1^NHS Greater Glasgow and Clyde Acute Division, Nursing, Midwifery and Community Health School, Glasgow Caledonian University, Govan Mbeki Building, Glasgow G4 0BA, UK; ^2^Golden Jubilee National Hospital, Beardmore Street, Clydebank G81 4HX, UK; ^3^College of Medical, Veterinary and Life Sciences, School of Medicine, Nursing and Health Care, University of Glasgow, 57-61 Oakfield Avenue, Glasgow G12 8LW, UK

## Abstract

*Objectives*. To evaluate changes in health related quality of life (HRQL) for individuals with Type 2 diabetes following the introduction of a new community-based model of care. *Methods*. A survey method was used in which HRQL, Problems Areas In Diabetes (PAID) and demographics were assessed before and 18 months after introducing the new service. *Results*. Overall HRQL and PAID scores were lower than published levels in individuals with diabetes but remained stable during the transition to the new model of care except for the bodily pain domain and deteriorating PAID scores for older patients. Four domains of SF36 health showed deterioration in the highest socio-economic groups. Deterioration was also observed in males, most notably mental health, in patients aged 54 years or less, 75 years or more and patients from socio-economic groups 1 and 2. HRQL was lowest at baseline and follow-up in socio-economic groups 6 & 7. Low levels of distress in patients across all deprivation categories was observed but remained stable over the transition. *Conclusions*. HRQL and distress associated with diabetes remained stable following the introduction of the new community-based model of care except for deterioration in the bodily pain domain and deteriorating PAID scores for older patients. *Relevance for Practice*. (i) Health related quality of life assessment is practical and acceptable to patients. (ii) In clinical governance terms it is good practice to monitor the impact of change in service delivery on the health of the patients in your care. (iii) Screening with health related quality of life tools such as generic and disease specific tools could help identify health problems otherwise undetected within current clinical care. Systematic identification of the most vulnerable groups with Type 2 diabetes should allow care to be better targeted.

## 1. Introduction

The increasing incidence of Type 2 diabetes globally is challenging to health care systems. Within the United Kingdom (UK), different models of care are proposed to cope with this challenge. 

In 2003, the Scottish Executive Health Department awarded *£*2.3 million to Greater Glasgow Health Board (GGHB), now defunct but at the time the largest Health Board in Scotland, to undertake a service redesign [1] aimed at meeting the requirements of policy documents, providing a service that is more accessible to people with Type 2 diabetes and consequently reducing morbidity and mortality associated with diabetes. An integrated model of care was proposed with general practitioners (GPs) based in primary care taking the lead role rather than hospital-based consultants in secondary care. Patients would be referred to secondary care based on clinical need. 

All members of the multiprofessional primary health care team were required to undergo accredited diabetes education. General Practices provided information technology (IT) and data management systems to support an annual review of clinical parameters and management of diabetes and risk factors. Additional new posts were created in community nursing, dietetics and podiatry to support the service redesign. 

The new model of care was based on the chronic care model [2]. This model focuses on six evidence-based areas of practice associated with improved outcomes in the management of patients with a chronic disease, namely, the community, the health care system, the design of the delivery system, the decision support system, the clinical information systems, and self-management support. The chronic care model [2] also suggests that informed and motivated patients in conjunction with prepared proactive teams can produce better care and improved outcomes [3]. This was a central tenet of the move to the new model of care. Key differences between the new and previous model are presented in Table 1.

Diabetes and its management can have a considerable impact on people's lives [4, 5], for example, feelings of isolation, codependency, experience of loss, overuse of defence mechanisms, and loss of freedom, all of which could have consequences for the optimal management of the condition.

The literature on the impact of a range of interventions to improve care for people with diabetes has produced conflicting findings. Some features of diabetes care and its management regimen have been shown to reduce HRQL [6]. On the other hand, specific improvements in quality of life have been reported when care was associated with regular clinical review (at least twice a year), continuity of care, education by the Diabetes Nurse and satisfaction with education [5]. Davies et al. [7] also found positive changes on depression scores, greater understanding of diabetes, perceived personal responsibility and weight loss following a structured education programme with 12-month followup. However, other authors who have evaluated specific educational interventions found that blood glucose monitoring or educational courses [8] had no impact on HRQL. 

HRQL is increasingly taken into account within health care provision as a measure of the effectiveness of care [9]. As part of a larger study, it was considered important to include not only clinical markers of effective service which are reported elsewhere [10], but also any potential impact on HRQL. 

This study was conducted to evaluate a new model of care for people with Type 2 diabetes and reports on the general health status and disease-specific health of individuals before and after the change in service delivery. 

### 1.1. Aims of This Study

The aim of the study is to assess HRQL for people within a defined geographical area who are experiencing a change in service delivery for their diabetes care management. Patterns of changes in HRQL were examined in relation to different age groups, gender, and socioeconomic deprivation categories.

## 2. Materials and Methods

### 2.1. Study Design

The before and after design of the study used validated and reliable questionnaires at baseline and followup 18 months after the implementation of the new service [10–13]. Two questionnaires were used as recommended in the literature [14]. Ethics permission was acquired from GGHB Primary Care Research Ethics Committee. 

#### 2.1.1. Questionnaires

**(a) Demographic Information**

Demographic details (age, sex, and postcode) were collected from the health care IT system and used to estimate socioeconomic status using an updated version of the Carstairs deprivation scores [15]. The deprivation score is based on vital statistics collected by UK Government surveys and is a number from 1 to 7 calculated from indicators such as lack of car ownership, male unemployment, postcode, and overcrowding, with 1 denoting the most affluent and 7 the most socioeconomically deprived.

**(b) General Health Status**

The health experiences of participants in the four weeks prior to assessment was measured using the SF-36 questionnaire [11]. The questionnaire itself consists of thirty-six questions measuring eight domains of health, namely, “physical functioning,” “role limitation due to physical” health problems, “bodily pain,” “general health,” “energy and vitality,” “social functioning,” “mental health,” and “role limitations due to mental” health problems. Each domain provides a score from 0 to 100 with zero indicating the worst health status and 100 the best. The questionnaire is based on a WHO definition of health, which states that health is not only defined by the absence of disease and infirmity, but also by the presence of physical, mental, and social well-being [16]. The domains themselves were developed in consultation with health professionals rather than patients. The scales were scored using a Likert's method of summated ratings. Each item was assumed to have a linear relationship with the score for its domain. The eight scales of the SF-36 questionnaire have been shown to have high internal consistency (Cronbach Alpha 0.76–0.86). Content validity (the extent to which SF-36 comprehensively measured health status) and criterion validity (the extent to which SF-36 correlated with existing measures of health) were established during this developmental stage. The SF-36 health assessment questionnaire has been reported as valid and reliable in normal populations as well as diabetes patient groups [14, 17, 18].

**(c) Diabetes Specific Emotional Distress**

The problem areas in diabetes (PAID) questionnaire is a reliable and valid tool to determine diabetes specific emotional distress [12, 13, 18]. It consists of 20 items measuring emotional adjustment to living with diabetes. These items are further constructed around the goals of treatment, family support, worry about complications, and eating and drinking. Each item is scored on a 5-point Likert scale according to the degree to which the individual perceives that it as a problem. Total scores vary between 0 and 100 with a higher score indicating greater emotional distress associated with diabetes.

### 2.2. Participants

At the time of the study, the primary care structure within GGHB was based on 14 local health care cooperatives (LHCCs), each of which was a functional unit for the delivery of care in a defined geographical area. The study was conducted within one LHCC with 14 GP practices and a registered patient population of 63,028 patients of whom 1,402 people were diagnosed with Type 2 diabetes. 

Every third person on each general practitioner's (GP) register with Type 2 diabetes was invited to take part in the study (*n* = 576). These individuals were sent a letter via their GP to inform them of the study, the nature of any participation and a written informed consent sheet granting permission to access their clinical records pertaining to diabetes care.

Consenting individuals were sent the SF-36 and the PAID questionnaires with instructions for their completion together with stamped addressed envelopes for their return at two time points. The questionnaires were completed in the first instance at the commencement of the new community-based model of care (2004) and again 18 months later (2006).

#### 2.2.1. Data Presentation and Analysis

Data are presented as mean ± standard deviation and median (interquartile range) for nonparametric distributions. Differences in the outcome variables were tested by comparison of baseline and follow-up data using *χ* ^2^ tests for categorical variables and Students' *t*-tests or Mann-Whitney tests for continuous variables (dependent upon data distribution) using Arcus Quickstat Biomedical software (Addison Wesley Longman trading as Research Solutions). The sample size allowed sufficient statistical power to detect a 10% change in the SF-36 scores with a confidence level of 90% and a *P* value of  .05.

## 3. Results

A total of 136 people were recruited to the study. Paired data for the questionnaire responses at baseline, and followup was available on 65% (*n* = 88) of the participants. 

Demographic details (age, gender, deprivation category, and ethnic origin) of the participants and the population group from which they were selected are presented in Table 2. Proportions (and mean/standard deviation for age) are calculated for the LHCC Type 2 diabetes population and for the sample of 136 participants. A Chi-squared test of equal proportions was used to compare our sample against the remaining LHCC patients. Significant differences are shown in bold. There is evidence of a difference in the distributions of age category, deprivation category, and ethnic origin between our sample and the rest of the LHCC. Unfortunately this means that it is not possible to state that the sample is representative of the full LHCC patient group at the time.

Scores from the SF-36 and the PAID questionnaires are presented in Tables 3, 4, and 5. Comparisons are made for the whole group between the SF-36 as applied to other diabetic patient groups and this sample (Table 3). Thereafter, SF-36 and PAID results are presented by gender, age categories and by deprivation category (Tables 4 and 5). 

### 3.1. SF-36 General Trends

Overall, the general health of the study group was similar to that of other published results on larger samples but tended towards reduced levels at followup. There was no evidence of “floor” or “ceiling” effects; that is, the scaling was sensitive at the extremes of the scales and could detect changes in states of very poor health and very good health. The “physical function,” “bodily pain,” “role limitation physical,” “role limitation mental,” and “social function” scores were all better than other published results for people with diabetes at the beginning of the change in service delivery, but there was a lowering of these scores over the course of the study. Scores in “general health,” “energy/vitality,” and “mental health” commenced at a lower level and remained lower (Table 3). However, scores for the whole group showed no statistically significant change at the *P* < .05 level after implementation of the new model of care, except in the “bodily pain” domain (*P* = .02).

There were no differences between genders with the exception of a deteriorating mental health score for males. The oldest group showed more deterioration in SF-36 scores across “physical function,” “role limitation due to physical function,” “energy and vitality,” and “bodily pain” (4 domains), whereas the youngest group showed more deterioration in “role limitation due to physical function,” “social function,” and “general health” (3 domains). There was also a statistically significant deterioration in “physical function,” “role limitation due to physical function,” “energy and vitality,” “bodily pain,” and “general health” (5 domains) for people in the highest socioeconomic groups (dep cat 1 & 2) with the scores for those in the other socioeconomic groups remaining stable (Table 4). HRQL was lower for all groups at baseline in socioeconomic groups 6 & 7 and remained so at follow-up assessment.

The middle years of 55–74 appear to be the most settled with no extremes in results. There was no evidence of improvement in SF-36 health domains. Any statistically significant changes across the whole group or within the patient subgroups examined were deterioration in health status.

### 3.2. PAID Scores

**Figure 1:**
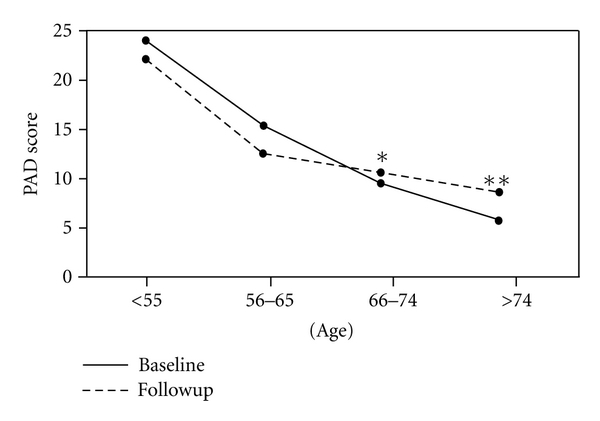
Baseline PAID score and follow-up PAID score versus age. *statistically significant difference with *P* = .045. **statistically significant difference with *P* = .020.

The results from this questionnaire were similar both before and after the implementation of the new community-based model of care (Figure 1). Scores indicated that there was sensitivity to different levels of distress associated with diabetes and were slightly lower than other published data for people with diabetes [13, 18] in respect of lower and unchanged levels of distress following the introduction of the new community-based service. However, scores were generally low (less distress), but were relatively higher in younger participants (Table 5) and exhibited statistically significant deterioration in patients aged 65 years or more (Figure 1).

## 4. Discussion

In the study group, no major differences were observed in the pattern of HRQL and PAID scores both by comparison with other published assessment [11] or over the timeframe of the introduction of the new service except in respect of a deterioration in the “bodily pain” domain of SF-36 which was due to other conditions unrelated to diabetes. However, a tendency towards lowering of health status over the period of the study (see results section) emerged. Data on the presence of other medical conditions was noted in participants' accounts of their health, for example, presence of arthritis, but this was not confirmed by checking medical records. 

An earlier study [19] showed no gender differences with the PAID questionnaire. This is further supported in the present study using the PAID and SF-36 questionnaires with the only exception being in respect of mental health for males. However, mental health scores were higher at baseline for male participants and higher than women overall. Nevertheless, the statistically significant result is that the mental health of males deteriorated during the period of the study, whereas that of females was stable.

This result contrasts with other work examining depression and diabetes which found no differences in levels of depression between men and women living with the condition [20]. This effect was observed only with the total group and was not obvious in analyses by age group or deprivation category. This was a surprise finding. Men are known to be less proactive than women in accessing health care facilities; nevertheless, there is no obvious reason why there should be such a deterioration in their mental health over this 18-month period.

A meta-analyses of studies examining links between depression in diabetes demonstrated a relationship with hyperglycaemia [21] and with an increased risk for complications [22]. People at elevated risk for depression can be identified through the medical history and clinical presentation and by asking depression-specific questions or through the use of depression screening tools. People with a history of depression, anxiety disorder, mental health treatment, substance abuse, or smoking are at heightened risk for depression, as are women and those with a family history of depression or mental health treatment. People who have multiple complications are more likely to be depressed, especially when those complications include neuropathy, impotence, or cardiovascular disease [22].

The older age group (>74years) experienced a greater deterioration in health scores than younger groups. This may reflect a group of people who are living with increased frailty generally but appear to have less psychosocial related health issues. By contrast, the youngest age group (<55years) had various deteriorations in health scores and, moreover, had the lowest, energy, and vitality scores for all age groups. A potential explanation for this observation is that people at this stage of life have many competing demands on time, for example, employment, commitments to children, and ageing parents although the study provides no supporting evidence.

Interestingly, the deterioration in health status as measured by the SF 36 questionnaire was statistically significant in deprivation categories 1 & 2 for various domains (Table 4). It is not clear why scores in this group decreased but the outcome are scores that are similar to participants from deprivation categories 3, 4, and 5. By implication this places this group of people at higher risk of increased mortality and morbidity associated with increased levels of socioeconomic deprivation. It would appear that those in deprivation categories 1 & 2 are experiencing HRQL issues as if they lived in a lower deprivation category. This was a surprise finding, implying that the more articulate, educated people are just as much in need of support as those from a more deprived background. 

It has been reported in the literature that diabetes disproportionately affects socially and materially disadvantaged individuals [23]. Higher levels of retinopathy, heart disease, and HbA1c and less health checks for the quality indicators of diabetes care have been reported leading to increased mortality and morbidity [24]. Our findings are similar in that we found the lowest levels of health scores in participants from the areas of highest socioeconomic deprivation. For participants from deprivation categories 3, 4, 5, and 6 & 7 there was a general deterioration in scores, but the trend was not statistically significant. This pattern of HRQL change differs from reports examining the impact of a range of interventions including diabetes education and behavioural modification (15 studies), pharmacotherapy (11 studies), and surgery (7 studies) in that these interventions generally demonstrated improvement in HRQL although the magnitude of effect varied [25]. Our evaluation is different in the respect that it assesses differences in two models of routine clinical care and as such the fundamental principles of care may not be radically changed compared with the testing of an additional focussed intervention. Recommendations have been made to develop further focussed strategies aimed at reducing inequalities of health outcomes for people with diabetes from areas of socioeconomic disadvantage [24].

It is interesting to note that PAID scores were at the lower level of distress severity compared with the generic SF-36 health domains where some domains were less than 50% of the possible optimal score. The statistically significant deterioration in scores in the older-age groups (Figure 1) could be related to the fact that these people had lived with the condition for a longer period of time and may be experiencing some of the wider pathological effects of diabetes. Alternatively, people newly diagnosed with diabetes in that age group may find the impact of diabetes greater than younger people. This conclusion, however, must be treated with caution, as it is based on the difference between just one individual.

A review of assessment and measurement of quality of life in people with Type 2 diabetes [26] acknowledged that there are many other variables that impact on quality of life, for example, demographics, comorbidities and psychosocial factors. The effect of daily ongoing monitoring of diet, exercise, medication management, and glucose monitoring to achieve as closely as possible a nondiabetic metabolic state was recognized as having a major impact on peoples' lives. New models of service delivery, such as that described here, can best support care management and are likely to improve HRQL.

Using both the SF-36 and PAID questionnaires allows insight into the impact of diabetes to health alone and a holistic assessment of overall health status. These questionnaires provide appropriate tools to evaluate a service that is moving from a specialist model to enhanced generalist community-based model of care, the latter being noted by participants to be important to them [27]. Because the general health scores were disproportionately lower than the disease-specific scores, it could be argued that a general, holistic health care context is more fitting for these patient's health care needs. 

## 5. Conclusions

In the group studied, HRQL and distress associated with diabetes remained stable following the introduction of a change in the delivery of care from a hospital-based setting to a community model of care. The only statistically significant deterioration in HRQL was in bodily pain and was identified as due to other health conditions and unrelated to diabetes. 

Although it is recognised that many factors impact on HRQL it is noteworthy that for particular age groups, people from socioeconomic groups 1 & 2 and males had significant deterioration in certain domains of their HRQL. The reasons for these findings are beyond the scope of this study but will form the basis for further investigation. In agreement with the literature, it was noted that HRQL was lowest at baseline in socioeconomic groups 6 & 7 and remained so at follow-up assessment.

The study confirms the value of measuring HRQL for people with diabetes, living with a chronic long-term condition, to identify changes in status as a mechanism for understanding wider health issues and developing individualised strategies to improve care. The HRQL measures have been shown to identify subgroups of people whose health may be particularly affected by the impact of diabetes mellitus. Assessment of HRQL could be integrated into annual review assessments.

## Figures and Tables

**Table 1:** Comparisons of key differences in approaches between the secondary care focussed model of care to a community model of care for patients with Type 2 diabetes.

Parameter	Previous	New model
Setting of diabetes care	Hospital based (secondary care)	General practitioner surgery (Primary care)

Access to care	Mixed community/secondary care	Community

Structure	IT systems	IT systems to support annual review, recall, and management systems introduced

Care provided	Annual Screening and review of clinical parameters	Annual screening and review of clinical parameters Followup appointments for management of clinical parameters; greater empowerment of self-care

	Laboratory results sent onto GP with recommendations for action	GP receives laboratory results directly and acts accordingly

	Recommendations to GP for change in prescriptions	GP alters prescriptions and initiates necessary therapies

	Review by dietitian, podiatrist, and diabetes specialist nurse at annual review that may require considerable waiting times	Review by dietitian, podiatrist, and practice nurse at annual review as part of a one stop shop so no waiting between professionals

	Management of diabetes and related risk factors	Management of diabetes, related risk factors within a holistic context

	Referral to specialist services as required for example, renal physicians	Referral to specialist services as required for example, renal physicians

Educational preparation	Staff have significant clinical expertise in diabetes with or without recognised qualifications	Staff all required to undertake a credit-rated qualification in diabetes

Retinal screening	Secondary care	National level directed

**Table 2:** Comparison of patient demographic details between the research sample and the local health cooperative (LHCC) population of patients with Type 2 diabetes*.

Patient numbers with Type 2 diabetes in the LHCC and the research sample	Total patient population in the LHCC with Type 2 diabetes	Research sample	LHCC patient population with Type 2 diabetes (excluding research sample)	*P*-value
Total Number	1402	136	1266	—

Gender				.10
Female	662 (47%)	55 (40%)	607 (48%)	
Male	740 (53%)	81 (60%)	659 (52%)	

Mean age (SD dev) in years	63.76 (13.59)	65.38 (11.96)	63.57 (13.75)	.08

Age Category				**.003**
<55 yrs	346 (25%)	28 (21%)	318 (25%)	
55–64 yrs	324 (23%)	23 (17%)	303 (24%)	
65–74 yrs	415 (30%)	56 (41%)	357 (28%)	
>74 yrs	317 (23%)	29 (21%)	288 (23%)	

Deprivation category				**.007**
1	38 (3%)	3 (2%)	35 (3%)	
2	260 (19%)	27 (20%)	233 (18%)	
3	160 (11%)	26 (19%)	134 (11%)	
4	252 (18%)	32 (24%)	220 (17%)	
5	85 (6%)	8 (6%)	77 (6%)	
6	187 (13%)	11 (8%)	176 (14%)	
7	420 (30%)	29 (21%)	391 (31%)	

Grouped dep cat				**.001**
1 and 2	268 (21%)	30 (22%)	268 (21%)	
3, 4 & 5	431 (34%)	66 (49%)	431 (34%)	
6 & 7	567 (45%)	40 (29%)	567 (45%)	

Ethnic origin				**<.001**
Asian	254 (18%)	8 (6%)	246 (19%)	
Other	1148 (82%)	128 (94%)	1020 (81%)	

*Chi Squared statistics were used to compare differenced in frequencies.

**Table 3:** Health-related quality of life scores: SF-36 by whole group and by gender before and 18 months after introduction of the new community model of care for people with Type 2 diabetes compared with other diabetic population groups (12).

SF-36 domains	SF 36 scores (9)	Whole group	Males	Females
*n*	541	88	52	36

Baseline/followup		B F	B F	B F

Physical function	67.7	75 versus 70	75 versus 78	73 versus 63
		*P* = .10	*P* = .24	*P* = .25

Role limitation physical	56.8	75 versus 50	100 versus 75	50 versus 25
		*P* = .08	*P* = .20	*P* = .24

Role limitation mental	75.6	88 versus 100	100 versus 100	67 versus 100
		*P* = .86	*P* = .82	*P* = .60

Social function	82.0	89 versus 78	89 versus 78	78 versus 78
		*P* = .07	*P* = .08	*P* = .48

Mental health	76.8	72 versus 70	75.4 versus 71.2	66.1 versus 67.7
		*P* = .18	*P* = .02	*P* = .46

Energy/vitality	55.7	51 versus 49	54.1 versus 49.9	47.5 versus 47.4
		*P* = .21	*P* = .09	*P* = .97

Bodily pain	68.5	70 versus 63.9	72.5 versus 67.4	67.3 versus 59.0
		*P* = .02	*P* = .12	*P* = .08

General health	56.1	54.8 versus 51.7	55.8 versus 51.1	53.4 versus 52.6
		*P* = 0.13	*P* = .09	*P* = .80

**Table 4:** Health-related quality of life scores: SF-36 before and 18 months after introduction of the new community model of care for people with Type 2 diabetes by age groupings and deprivation category. Statistical comparisons of baseline and follow-up data, when these data are not normally distributed, are based on the Mann-Whitney-Wilcoxon rank test.

SF-36 domains	Age <55 years	Age 55–64 years	Age 65–74 years	Age >74 years	Dep cat 1&2	Dep cat 3, 4 & 5	Dep cat 6 & 7
*n*	15	17	39	17	24	41	23

Baseline/followup	B F	B F	B F	B F	B F	B F	B F

Physical function	75 versus 80 (55–100)	75 versus 80 (43–90)	75 versus 70 (50–90)	65 versus 45 (45–78)	75 versus 75	75 versus 75	50 versus 45
	*P* = .06	*P* = .19	*P* = .40	*P* < .001	*P* = .04	*P* = .23	*P* = .64

Role limitation physical	100 versus 50 (0–100)	100 versus 100 (13–100)	50 versus 50 (0–100)	50 versus 0 (13–100)	100 versus 50	100 versus 100	25 versus 0
	*P* = .045	*P* = .29	*P* = .51	*P* = .013	*P* = .01	*P* = .92	*P* = .57

Role limitation mental	100 versus 33 (0– 100)	100 versus 100 (46–100)	67 versus 100 (33–100)	100 versus 67 (17–100)	100 versus 100	75 versus 100	57 versus 33
	*P* = .57	*P* = .46	*P* = .81	*P* = .52	*P* = .80	*P* = .25	*P* = .22

Social function	89 versus 67 (33–100)	89 versus 78 (56–100)	89 versus 89 (67–100)	78 versus 67 (62–95)	100 versus 89	89 versus 89	67 versus 56
	*P* = .006	*P* = .54	*P* = .71	*P* = .10	*P* = .06	*P* = .88	*P* = .11

Mental health	59.5 versus 56.3	66.8 versus 67.5	75.3 versus 73.6	78.6 versus 74.8	81.0 versus 77.5	70.7 versus 70.6	63.3 versus 60.0
	*P* = .41	*P* = .71	*P* = .47	*P* = .13	*P* = .06	*P* = .96	*P* = .35

Energy/vitality	41.7 versus 41.3	52.4 versus 52.4	52.1 versus 51.2	57.7 versus 46.8	62.7 versus 54.4	51.1 versus 50.2	40.2 versus 40.7
	*P* = .92	*P* = 1.00	*P* = .73	*P* = .04	*P* = .02	*P* = .74	*P* = .93

Bodily pain	68.3 versus 57.9	66.1 versus 68.1	70.4 versus 65.3	76.5 versus 62	79.3 versus 67.6	70.5 versus 67.3	60.9 versus 54.2
	*P* = .19	*P* = .74	*P* = .18	*P* = .03	*P* = .03	*P* = .35	*P* = .30

General health	50.3 versus 41.4	52.3 versus 55.8	56.7 versus 54.0	56.8 versus 51.5	64.1 versus 55.1	55.6 versus 53.6	43.6 versus 44.7
	*P* < .001	*P* = .48	*P* = .46	*P* = .13	*P* = .02	*P* = .40	*P* = .83

**Table 5:** Health-related quality of life scores: PAID questionnaires before and 18 months after introduction of community model of care for people with Type 2 diabetes.

Domain	Whole group	Males	Females	Age <55 years	Age 55–64 years	Age 65–74 years	Age >74 years	Dep cat 1 & 2	Dep cat 3, 4 & 5	Dep cat 6 & 7
*n*	88	52	36	15	17	39	17	24	41	23

Baseline/ followup	B F	B F	B F	B F	B F	B F	B F	B F	B F	B F

PAID	13 versus 13	12 versus 13	13 versus 13	25 versus 23	16 versus 13	10 versus 11	6 versus 9	9 versus 9	13 versus 14	14 versus 13
	*P* = .14	*P* = .17	*P* = .55	*P* = .86	*P* = .39	*P* = .045	*P* = .02	*P* = .11	*P* = .44	*P* = .54
